# Mouse models of preeclampsia with preexisting comorbidities

**DOI:** 10.3389/fphys.2023.1137058

**Published:** 2023-04-06

**Authors:** Christopher A. Waker, Amy E. Hwang, Scout Bowman-Gibson, Chandni H. Chandiramani, Bryce Linkous, Madison L. Stone, Chanel I. Keoni, Melissa R. Kaufman, Thomas L. Brown

**Affiliations:** ^1^ Department of Neuroscience, Cell Biology and Physiology, Boonshoft School of Medicine, Wright State University, Dayton, OH, United States; ^2^ Department of Obstetrics and Gynecology, Boonshoft School of Medicine, Wright State University, Dayton, OH, United States

**Keywords:** preeclampsia, diabetes, obesity, hypertension, comorbidity, mouse model

## Abstract

Preeclampsia is a pregnancy-specific condition and a leading cause of maternal and fetal morbidity and mortality. It is thought to occur due to abnormal placental development or dysfunction, because the only known cure is delivery of the placenta. Several clinical risk factors are associated with an increased incidence of preeclampsia including chronic hypertension, diabetes, autoimmune conditions, kidney disease, and obesity. How these comorbidities intersect with preeclamptic etiology, however, is not well understood. This may be due to the limited number of animal models as well as the paucity of studies investigating the impact of these comorbidities. This review examines the current mouse models of chronic hypertension, pregestational diabetes, and obesity that subsequently develop preeclampsia-like symptoms and discusses how closely these models recapitulate the human condition. Finally, we propose an avenue to expand the development of mouse models of preeclampsia superimposed on chronic comorbidities to provide a strong foundation needed for preclinical testing.

## Introduction

Preeclampsia is a life-threatening condition that complicates 2%–7% of all pregnancies (Roberts and Lain, 1998; Sibai, 2006; Abalos et al., 2013; Albers et al., 2019; Poon et al., 2019; Chappell et al., 2021; Waker et al., 2021). It is one of the leading causes of maternal and fetal morbidity and mortality (Poon et al., 2019; Chappell et al., 2021). The pathological features of preeclampsia were historically defined as rapid-onset, pregnancy-specific hypertension with accompanying proteinuria and parturitional resolution. Recent reclassification, however, now includes maternal renal, hepatic, pulmonary, or neurological involvement in the absence of proteinuria (Rana et al., 2019; Obstetrics and Gynecology, 2020; Garovic et al., 2022). Abnormal placental development or dysfunction is thought to be the root cause of the condition, as the only known cure is delivery of the placenta (Hladunewich et al., 2007; Roberts and Escudero, 2012; Ilekis et al., 2016; Schneider, 2017).

**FIGURE 1:**
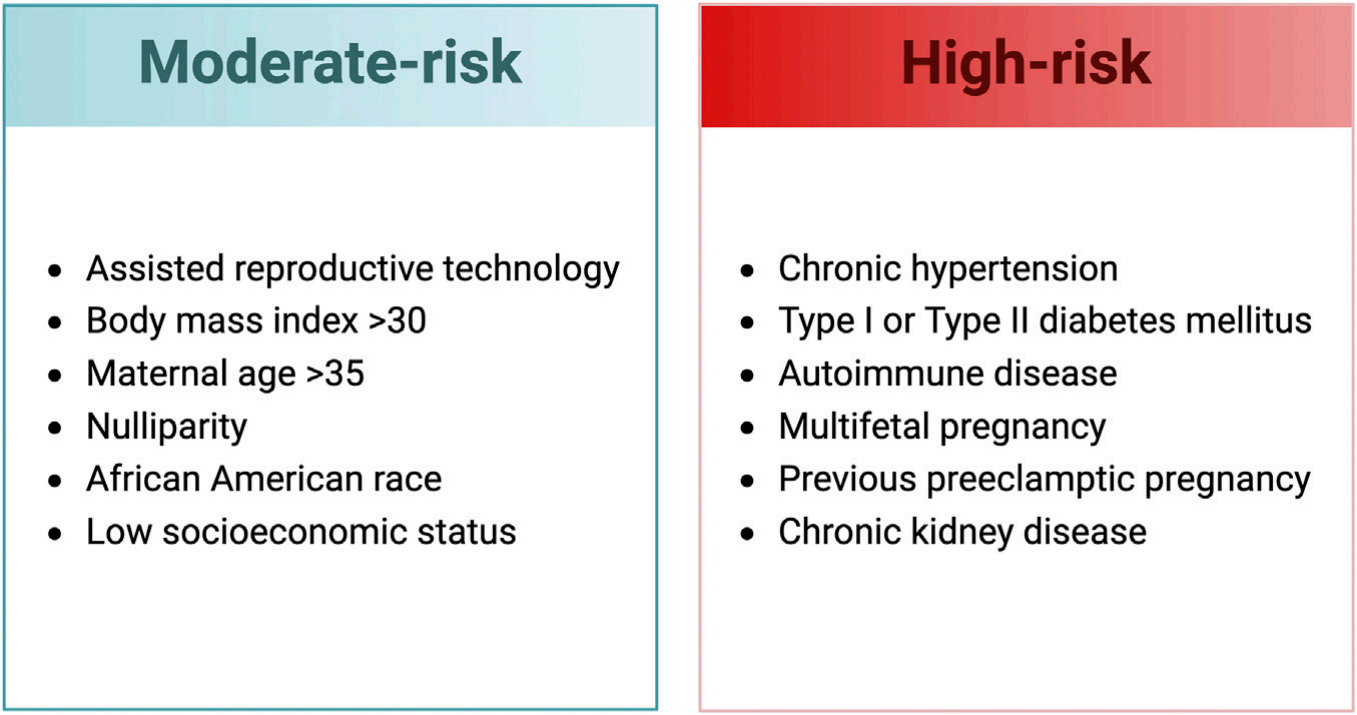
Clinical risk factors for the development of preeclampsia (SMFM Patient Safety and Quality Committee et al., 2020; Obstetrics and Gynecology, 2020). Created with BioRender.com.

While preexisting comorbidities are a common occurrence in preeclamptic pregnancies, there is a paucity of information on how they impact the development, progression, or severity of the condition. Clinically, preexisting conditions such as prior preeclampsia, chronic hypertension or kidney disease, pregestational diabetes, and autoimmune disease are associated with a high risk of developing preeclampsia (Figure 1). Why women with certain comorbidities have an increased risk of preeclampsia is unknown and it is unclear if specific treatment plans should be implemented based on a particular comorbidity. Preeclamptic pregnancies associated with comorbidities have a higher rate of caesarian section birth and are delivered earlier than preeclampsia pregnancies that do not have preexisting comorbidities (Tanner et al., 2022). In addition, neonates from preeclamptic pregnancies that have preexisting comorbidities also experience an increase in respiratory distress syndrome, neonatal sepsis, and neonatal intensive care unit admissions (Tanner et al., 2022).

Recent reports have identified early-onset (<34 weeks) or late-onset (>34 weeks) as distinct subtypes of preeclampsia, based on the gestational time of diagnosis (Raymond and Peterson, 2011; Roberts and Escudero, 2012; Redman et al., 2014; Gathiram and Moodley, 2016; Staff and Redman, 2018; Waker et al., 2021). This raises the possibility that preeclampsia may have distinct subtypes due to the influence of the preexisting comorbid condition. As the prevalence of obesity, chronic hypertension, and diabetes continues to increase worldwide, understanding how these comorbidities increase the risk of developing preeclampsia is paramount to understanding the genesis of the condition and developing effective treatment regimens.

Mouse models are a widely used and well-accepted tool to study preeclampsia because of their extensive genetic characterization and high homology to humans, as well as similar hemochorial blood flow (Maltepe and Fisher, 2015; Soncin et al., 2015; Soares et al., 2018; Hu and Zhang, 2021; Waker et al., 2021). To investigate the effects of preexisting comorbidities on the development of preeclampsia, mouse models have been developed using gene knockout, transgenic overexpression, dietary supplementation, and selective inbreeding. The goal of this review is to provide researchers with an analysis of mouse models that have preexisting conditions and develop preeclampsia-like symptoms during pregnancy (Table 1).

**TABLE 1:** Current mouse models with preexisting conditions that develop PE.

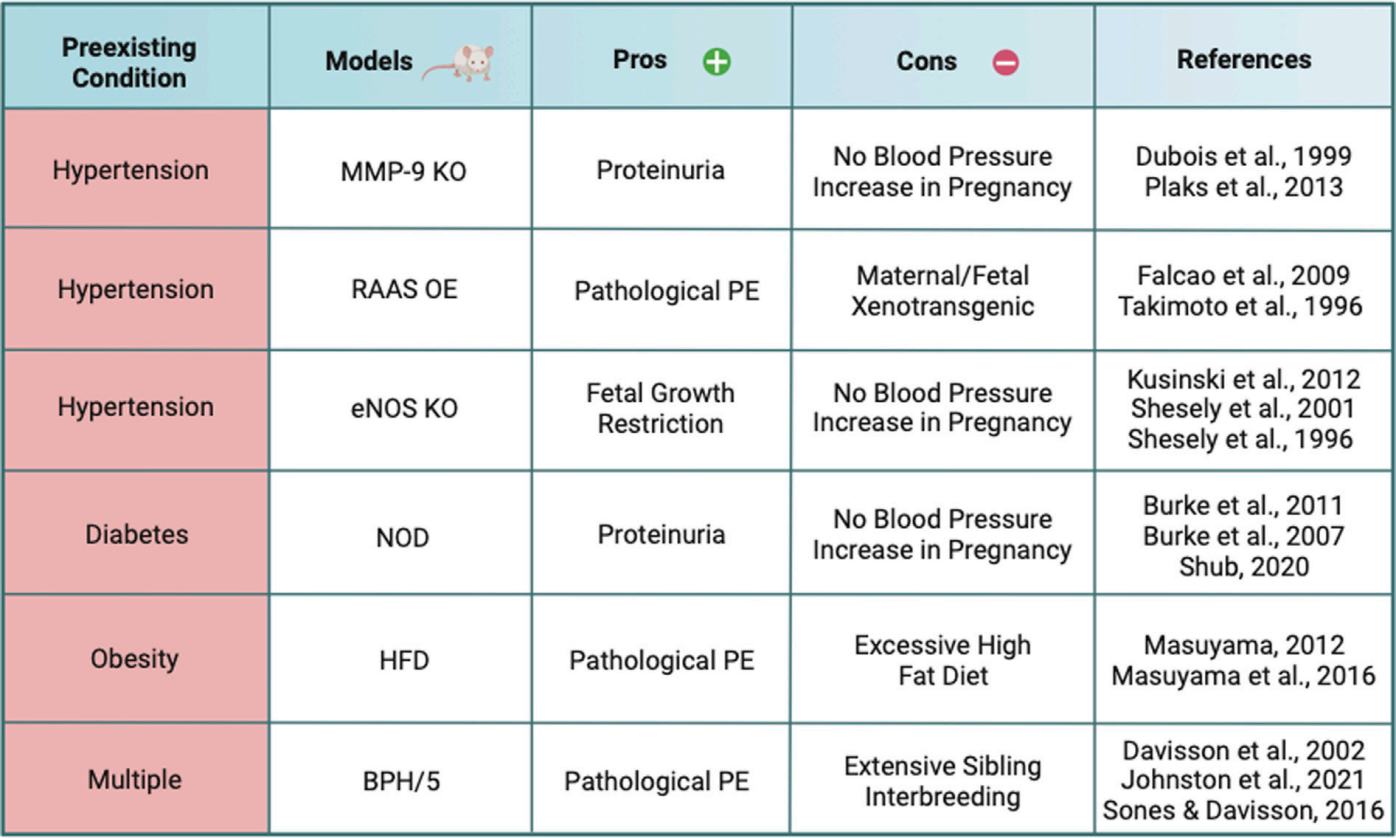

Advantages (Pros) and limitations (Cons) of mouse models with preexisting conditions that exhibit pathological features of preeclampsia, namely, hypertension and proteinuria. Abbreviations used: PE (preeclampsia), MMP-9 (matrix metalloproteinase-9), KO (knockout), RAAS (renin-angiotensin-aldosterone system), eNOS (endothelial nitric oxide synthase), NOD (non-obese diabetic), HFD (high fat diet), BPH/5 (blood pressure high/5). Created with BioRender.com.

## Chronic hypertension

In the United States, chronic hypertension affects 45% of adults and up to 10% of all pregnancies (Muntner et al., 2018; Topel et al., 2018; Battarbee et al., 2020). Patients with chronic hypertension have an increased risk of adverse health conditions such as coronary artery disease, stroke, heart failure, and renal disease (Elliott, 2007). Preexisting hypertension is associated with additional complications during pregnancy such as preterm birth, fetal growth restriction, and preeclampsia; with preeclampsia being the most prevalent (Seely and Ecker, 2014). Chronic hypertension is high blood pressure that occurs prior to pregnancy or before 20 weeks of gestation. Preeclampsia is the rapid elevation of blood pressure, above preexisting stable hypertensive levels, after the 20th week of pregnancy accompanied with other systemic involvement.

A study of 822 pregnant women with chronic hypertension found that 22% of these patients developed preeclampsia (Chappell et al., 2008). Notably, nearly 50% of those with preexisting chronic hypertension developed early-onset preeclampsia (Chappell et al., 2008). In contrast, preeclampsia in the general population occurs in 2%–7% of pregnancies, with early-onset cases comprising about 16% (Abalos et al., 2013; Ananth et al., 2013; Lisonkova and Joseph, 2013; Fingar et al., 2017; Mayrink et al., 2019). The substantially increased prevalence of preeclampsia and early-onset cases in pregnancies complicated by chronic hypertension suggest that preexisting maternal hypertension is a significant predisposition in the development of the condition.

### Mouse models that eliminate matrix metalloproteinase-9 (MMP-9)

Matrix metalloproteinases comprise a family of zinc-dependent proteases that degrade extracellular matrices. Specifically, matrix metalloproteinase-9 (MMP-9) degrades type IV, V, and IX collagens, gelatin, and elastin and is involved in numerous processes, including implantation, placentation, and embryogenesis (Vu et al., 1998; Silvia and Serakides, 2016; Espino et al., 2017; Quintero-Fabián et al., 2019; Timokhina et al., 2020). During pregnancy, MMP-9 has been shown to be involved in endometrial remodeling and the invasion of placental extravillous trophoblasts (Zhang et al., 2020). Conversely, decreased levels of MMP-9 are associated with impaired trophoblast invasion (Staun-Ram et al., 2004; Chen and Khalil, 2017).

The data demonstrating an association of MMP-9 in human preeclampsia are variable. Some reports have shown reduced levels of circulating MMP-9 in maternal plasma from preeclamptic pregnancies; however, other studies have found elevated levels or no significant differences (Montagnana et al., 2009; Plaks et al., 2013; Eleuterio et al., 2015; Laskowska, 2017; Timokhina et al., 2020). In a study looking at the association of MMP-9 in severe preeclampsia, mild preeclampsia, and normal pregnancies; MMP-9 expression was reduced in severely preeclamptic patients, but was not different between mild preeclampsia and normal pregnancies (Wang et al., 2015; Zhang et al., 2019). Some of these discrepancies may be attributable to MMP-9 levels in pregnancy, as MMP-9 have been shown to be positively correlated with gestational age (Montagnana et al., 2009).

Dubois et al. initially reported impaired reproduction in MMP-9 null mice. In these mice, MMP-9 deficiency was associated with a decrease in the number of pregnancies as well as a reduction in litter size (Dubois et al., 1999; Dubois et al., 2000). Plaks and Rinkenburger also investigated the role of MMP-9 in pregnancy using MMP-9 deficient mice and found these mice are sub-fertile with decreased implantation and increased fetal demise (Plaks et al., 2013). In pure or mixed backgrounds with homozygous MMP-9 null crosses (129SV/J on CD1 or Swiss black backgrounds), there was up to a 20% reduction in litter size; however, on a C57BL/6J mouse background, MMP-9 null homozygous crosses exhibited a 50% reduction in litter size (Plaks et al., 2013). The variation in litter size among different strains of mice suggests that genetic background may be a contributing factor in the varying effects observed.

When embryos from MMP-9 null homozygous crosses were analyzed at E10.5, 18.4% exhibited fetal growth restriction when compared to heterozygous control. Additionally, of the embryos that were growth restricted, it was reported that they were in a “twisted” and “constrained” state. Homozygous MMP-9 knockout mice had reduced and malformed ectoplacental cones, surrounded by blood pools at E7.5, impaired trophoblast differentiation, and reduced invasion (Plaks et al., 2013). The MMP-9 null placentas exhibited altered morphology with an increased number of trophoblast giant cells and diminished spongiotrophoblast and labyrinth layers (Plaks et al., 2013). Interestingly, it was reported that normal placental development required both maternal and fetal MMP-9 expression (Plaks et al., 2013).

When compared to non-pregnant controls, non-pregnant homozygous MMP-9 knockout mice were reported to have an elevated mean systolic blood pressure (Plaks et al., 2013). Importantly, pregnant homozygous MMP-9 knockout mice with viable fetuses exhibited a prolonged decrease in blood pressure as gestation progressed, compared to controls (Plaks et al., 2013). Kidneys from pregnant and non-pregnant MMP-9 null homozygous mice were also assessed. During pregnancy, the percentage of “open” glomerular capillaries in MMP-9 null mice was significantly reduced and proteinuria was present, which may indicate a pathology similar to glomerular endotheliosis (Plaks et al., 2013). While MMP-9 knockout mice have preexisting hypertension, the use of these mice as a comorbid model that develops preeclampsia-like symptoms requires consideration due to the uncharacteristic lack of elevated blood pressure during pregnancy.

### Mouse models that overexpress factors in the renin-angiotensin-aldosterone system

The renin-angiotensin-aldosterone system (RAAS) maintains blood pressure by regulating sodium, potassium, and fluid volume. Dysregulation of the renin-angiotensin-aldosterone system leads to the development of chronic hypertension (Santos et al., 2012; Carey, 2015; Te Riet et al., 2015). Activation of renin-angiotensin-aldosterone system occurs when the precursor, prorenin, is converted to renin by the juxtaglomerular cells of the kidney (Hsueh & Baxter, 1991; von Lutterotti et al., 1994). Renin proteolytically cleaves angiotensinogen and subsequent processing results in the generation of angiotensin I and II. Angiotensin II, the predominant end-product of the renin-angiotensin-aldosterone system, binds to the angiotensin II type 1 receptor 1 (AT1) and results in the activation of downstream signaling pathways that ultimately lead to vasoconstriction and increased retention of sodium and water by the kidney (Spaan & Brown, 2012). Furthermore, angiotensin II stimulates release of aldosterone from the adrenal glands to promote blood volume expansion. The physiological response to vasoconstriction, sodium reabsorption, and volume expansion is increased blood pressure (Spaan & Brown, 2012).

In a normal healthy pregnancy, the levels of renin, angiotensinogen, angiotensin I and II, and aldosterone are elevated compared to non-pregnant women, but a vasodilatory state is present such that hypertension does not typically occur (Brown et al., 1963; Brown et al., 1997). The increased level of progesterone during pregnancy decreases the sensitivity to angiotensin II and changes the state of AT1 receptor binding so that twice as much angiotensin II is required to elicit an elevation in blood pressure that would typically occur while not pregnant (Assali & Westersten, 1961; Gant et al., 1973; AbdAlla et al., 2001). Thus, increased levels of angiotensin II are required during pregnancy to maintain a normotensive blood pressure (Irani & Xia, 2008). In patients with preeclampsia; however, the maternal levels of plasma renin, angiotensin II, and aldosterone have been shown to be lower than normotensive pregnancies (Weir et al., 1973; Gant et al., 1980; Brown et al., 1997; Irani and Xia, 2008; Leaños-Miranda et al., 2018).

Falcao et al., sought to investigate the occurrence of preeclampsia-like features in a hypertensive mouse model during pregnancy using double transgenic mice that overexpress the genes for human renin (REN, R+) and human angiotensinogen (AGT, A+) (R+A+ mice) (Sigmund et al., 1992; Falcao et al., 2009). Non-pregnant R+A+ mice have significantly elevated angiotensin II and mean arterial pressure compared to non-transgenic, non-pregnant control mice (Falcao et al., 2009). Pregnant R+A+ mice had significantly elevated blood pressure above mean arterial pressure levels on gestational days 5 and 17, compared to non-pregnant R+A+ mice (Falcao et al., 2009). The mean arterial pressure at gestational day 18 for R+A+ mice was significantly higher than non-transgenic mice, indicating that in R+A+ mice, pregnancy further exacerbates their hypertensive state (Falcao et al., 2009). The R+A+ mice MAP decreased 24 h after birth but did not completely return to prepregnant levels.

Pregnant R+A+ mice developed proteinuria by the end of gestation but did not have signs of glomeruloendotheliosis or other renal pathology (Falcao et al., 2009). In addition, pregnant and non-pregnant R+A+ mice exhibited cardiac hypertrophy. Placental pathology included increased necrosis and loss of labyrinthine trophoblast structure. Although no differences in litter size were observed, fetal and placental weights were both significantly reduced, compared to non-transgenic mice.

The Falcao et al. model is similar to one generated by Takimoto et al., who analyzed AGT overexpressing female mice crossed with REN overexpressing male mice (Takimoto et al., 1996). Takimoto reported that female AGT mice became hypertensive after day 14 of gestation and had glomerular enlargement and increased urinary protein, coinciding with the development of the placental renin overexpression. This indicates that these transgenic mice exhibit a pregnancy-specific increase in blood pressure, a hallmark of preeclampsia. These pregnant mice also developed myocardial concentric hypertrophy with only 38% surviving pregnancy (Takimoto et al., 1996). In addition, 15% of these transgenic hypertensive mice had generalized convulsions late in pregnancy. Placental analysis indicated necrotic cell death in the spongiotrophoblasts and decidual cells and chorionic congestion (Takimoto et al., 1996).

Both models show that the excess secretion of renin and other renin-angiotensin-aldosterone system proteins or angiotensin-like peptides by the placenta can lead to the development of preeclampsia-like symptoms during mouse pregnancy (Takimoto et al., 1996; Shah et al., 2000; Falcao et al., 2009; Denney et al., 2017). The paradox between overexpression of renin and angiotensinogen creating preeclampsia-like symptoms in mice and the reduced levels observed in humans with preeclampsia suggests that renin-angiotensin-aldosterone system may be a downstream effect of other dysregulated systems. Additionally, most currently available therapeutic inhibitors of renin-angiotensin-aldosterone system are teratogenic and fetotoxic, precluding their use to treat preeclampsia (Alwan et al., 2005; Alwasel et al., 2010; Ferreira et al., 2010).

### Mouse models that eliminate endothelial nitric oxide synthase (ENOS)

Nitric oxide synthases [NOS I (nNOS), NOS II (iNOS), and NOS III (eNOS)] are a family of enzymes that create nitric oxide (NO) *via* the reduction of L-arginine to L-citrulline (Moncada and Higgs, 1993; Hefler et al., 2001; Förstermann and Sessa, 2012; Qian and Fulton, 2013). In the endothelium, the primary function of NO is to relax vascular smooth muscle tissue and it serves as an important regulator of arterial blood pressure (Shesely et al., 2001; Endres et al., 2004; Chen and Zheng, 2014; Guerby et al., 2021). Nitric oxide and the NOS enzymes play an important role in cardiovascular remodeling during development and are involved in the synthesis of vascular endothelial growth factor (VEGF), stimulation of endothelial progenitor cell activity, and angiogenesis *via* hypoxia inducible factor 1 alpha (HIF-1α) (Shesely et al., 2001; Endres et al., 2004; Chen and Zheng, 2014; Guerby et al., 2021). In a healthy normotensive pregnancy, nitric oxide levels spike early in gestation and gradually increase through the third trimester (Owusu Darkwa et al., 2018). In contrast, the levels of nitric oxide reported in preeclamptic women are conflicting; with reports showing increased, decreased, or no differences, compared to control (Seligman et al., 1994; Lyall et al., 1995; Smárason et al., 1997; Choi et al., 2002; Marshall et al., 2018).

Several groups have investigated the effect of eNOS knockout on blood pressure and the generation of preeclampsia-like symptoms in mice, following reports that chronic non-specific pharmacologic inhibition of NOS recapitulates some symptoms of preeclampsia (Baylis & Engels, 1992; Yallampalli & Garfield, 1993; Shesely et al., 1996; Hefler et al., 2001; Shesely et al., 2001; Kusinski et al., 2012). Three independent studies have reported that non-pregnant eNOS knockout mice have elevated blood pressure compared to controls (Huang et al., 1995; Shesely et al., 1996; Kusinski et al., 2012).

Hefler et al. examined pregnant eNOS homozygous knockout mice and reported reduced fetal weights; however, no placental abnormalities were noted. In that study, blood pressure and proteinuria were not reported, but severe limb abnormalities were identified (Hefler et al., 2001). Shesely et al., found that non-pregnant homozygous eNOS knockout mice had significantly increased blood pressure compared to control mice; however, blood pressure did not significantly increase during pregnancy (Shesely et al., 1996; Shesely et al., 2001). Similarly, Kusinski et al., observed that eNOS knockout mice had elevated blood pressure before and during pregnancy at gestational day 17.5, when compared to wild type mice (Kusinski et al., 2012). However, there was no significant increase in blood pressure between pregnant eNOS knockout mice and non-pregnant eNOS knockout mice (Kusinski et al., 2012). Investigations into placental structure determined that spiral artery remodeling was dysregulated, the labyrinth zone was reduced, uteroplacental hypoxia was present, and placental nutrient transport was reduced in eNOS knockout mice (Kulandavelu et al., 2012; Kusinski et al., 2012; Kulandavelu et al., 2013). Levels of VEGF were reduced and HIF-1α protein was increased in eNOS knockout placentas; whereas, levels of souble fms-like tyrosine kinase-1 (sFLT-1) in maternal plasma were not different between eNOS knockout and control (Kulandavelu et al., 2012; Kusinski et al., 2012; Kulandavelu et al., 2013).

Litter size was also reduced in eNOS knockout compared to control mice (Kulandavelu et al., 2013). Additionally, maternal and fetal weights were reduced in eNOS knockout mice compared to control at gestational day 17.5, but placental weights were not different (Kulandavelu et al., 2012; Kusinski et al., 2012; Kulandavelu et al., 2013). eNOS null mice have consistently been reported to be small and could serve as a model of fetal growth restriction; however, these mice have not been shown to exhibit a significant elevation in blood pressure during gestation that is characteristic of the preeclamptic condition (Medica et al., 2007; McCarthy et al., 2011; Kulandavelu et al., 2012; Kusinski et al., 2012; Qi et al., 2013; Alpoim et al., 2014).

## Diabetes

Type 1 diabetes mellitus is an autoimmune disease that causes endogenous insulin insufficiency (Chen et al., 2018; Sugrue and Zera, 2018). Type 2 diabetes mellitus is generally characterized by increased insulin resistance, rather than insulin insufficiency, as a result of chronically elevated serum levels of glucose and triglycerides (Salzer et al., 2015; Sugrue and Zera, 2018; Echeverria et al., 2020). In an uncomplicated pregnancy, peripheral insulin resistance increases approximately three-fold, compared to the non-pregnant state, in order to accommodate the developing fetus (Salzer et al., 2015; Kanasaki, 2018). This insulin resistance and subsequent hyperglycemic state create a diabetogenic environment (Kanasaki, 2018). Individuals with preexisting diabetes, however, have insufficient production or an inadequate response to insulin, thus impairing their ability to adapt to the metabolic demands of pregnancy.

Diabetes mellitus is strongly associated with the development of preeclampsia (Scioscia et al., 2009; Burke et al., 2011; Gutaj et al., 2017; Sugrue and Zera, 2018). Pregnancies complicated by pregestational Type 1 diabetes mellitus developed preeclampsia in 18% of cases, compared to 2.6% in the control population identified in this study (Jensen et al., 2004). Additional studies have reported that 11% of pregnancies with preexisting Type 2 diabetes later developed preeclampsia (Jensen et al., 2004; Groen et al., 2013). How pregestational Type 1 and Type 2 diabetes increase the risk of developing preeclampsia is not well understood and requires further investigation.

### Mouse models of type 1 diabetes mellitus

Non-obese diabetic (NOD) mice are an inbred strain characterized by the spontaneous development of autoimmunity and Type 1 diabetes mellitus. These mice have a mutation that results in depletion of regulatory T-cells and leads to the death of the pancreatic islet beta cells (D’Alise et al., 2008). NOD mice are widely used to study the pathophysiology of Type 1 diabetes mellitus, as they demonstrate autoimmune cell infiltration into pancreatic islet beta cells characteristic of the disease (Chen et al., 2018). Notably, not all non-obese diabetic mice will develop hyperglycemia in their lifetime, as the degree of pancreatic immune cell infiltration determines whether NOD mice will develop diabetes (Chen et al., 2018). Non-obese diabetic mice with mild pancreatic autoimmune cell infiltration can maintain normal blood glucose levels and never progress to a Type 1 diabetic phenotype; whereas, non-obese diabetic mice that have extensive insulitis develop a phenotype more severe than what occurs in the human condition. Nevertheless, the NOD mouse model most closely mirrors the spontaneous onset of Type 1 diabetes mellitus (Burke et al., 2011; Chen et al., 2018).

Pregnant non-obese diabetic mice are reported to have significantly increased proteinuria as gestation progresses with accompanying renal histopathology indicative of acute kidney injury (Burke et al., 2011). Pregnant non-obese diabetic mice also exhibit progressive bradycardia and reduced blood pressure from gestational day 10 through gestational day 18, compared to pregnant non-obese nondiabetic mice (Burke et al., 2011). The placentas in pregnant non-obese diabetic mice demonstrated impaired spiral artery remodeling, as a reduction in the number of spiral arteries and decreased luminal diameters (Burke et al., 2007; Burke et al., 2011). Placental weights from non-obese diabetic pregnancies were significantly increased; whereas, pup weights at birth were significantly lower compared to non-diabetic controls (Burke et al., 2007). Pregnant non-obese diabetic mice do exhibit kidney dysfunction and proteinuria in the diabetic animals, compared to the non-diabetic non-obese diabetic mice; however, the variability in the generation of a diabetic phenotype and unexpected cardiac features indicate further work is needed to model this comorbidity. Additionally, the decrease in blood pressure in pregnant non-obese diabetic mice is not representative of the preeclamptic condition in humans with pregestational Type 1 diabetes mellitus (Burke et al., 2011; Shub, 2020; Shub and Lappas, 2020).

## Obesity

The prevalence of obesity has reached epidemic proportions (Barnes, 2011; Olson et al., 2019). Obesity is a condition in which an individual has excess adipose tissue (body fat) and is clinically characterized by body mass index (BMI). This condition is associated with low grade inflammation and is often accompanied by dyslipidemia, decreased insulin sensitivity, and cardiovascular disease; collectively known as metabolic syndrome (Roberts et al., 2011; Kim et al., 2014; Howell and Powell, 2017; Olson et al., 2019). In particular, obesity has dramatically increased in women by more than 65% over the last 40 years (Wang and Beydoun, 2007; Barnes, 2011; Jeyabalan, 2013; Olson et al., 2019). According to the National Institute for Diabetes and Digestive and Kidney Diseases, the incidence of overweight (BMI: 25–29.9) and obese (BMI: ≥30) adult females in the United States is a striking 67% (Flegal et al., 2010; Fryar et al., 2020). Obesity is the leading risk factor for the development of Type II diabetes (Barnes, 2011).

Obesity is associated with an increased risk of developing complications during gestation and has been reported to be present in ∼30% of all pregnancies in the United States (Roberts et al., 2011; Driscoll and Gregory, 2020). Obesity during pregnancy is associated with numerous complications such as gestational hypertension, gestational diabetes, preterm birth, stillbirth, and preeclampsia (Yogev and Catalano, 2009; Roberts et al., 2011; Myatt and Maloyan, 2016; Lopez-Jaramillo et al., 2018; Kelly et al., 2020; Dumolt et al., 2021). Additionally, maternal obesity is associated with alterations in fetal weight, as neonates have an increased risk for growth restriction, but more commonly, macrosomia (Ornoy, 2011; Kanda et al., 2012; Tenenbaum-Gavish and Hod, 2013). The impact of obesity on fetal development can lead to potential life-altering cardiovascular, metabolic, and neurocognitive conditions for offspring later in life (Howell and Powell, 2017; Cirulli et al., 2020; Kelly et al., 2020; Dumolt et al., 2021).

Human and animal studies have reported that obesity can alter placental function (Kim et al., 2014; Saben et al., 2014; Spradley et al., 2015a; Spradley et al., 2015b; Olson et al., 2019; Wallace et al., 2019; Hoch et al., 2020). Placental expression of glucose, fatty acid, and amino acid transporters has been shown to be increased in obese individuals during human pregnancy and increased expression of placental nutrient transporters is strongly correlated with fetal birth weight (Jansson et al., 2013; Acosta et al., 2015; Lager et al., 2016; Howell and Powell, 2017; Vaughan et al., 2021). Maternal obesity may also lead to excess lipid accumulation in the placenta and could potentially interfere with trophoblast invasion, nutrient transport, and angiogenesis, which are often affected in preeclampsia (Saben et al., 2014).

The risk of developing preeclampsia during pregnancy is strongly correlated with maternal prepregnancy body mass index, with the risk doubling for overweight individuals and tripling for those clinically defined as obese (Bodnar et al., 2005; Robillard et al., 2019). While obesity is clearly indicated as a risk factor for the development of preeclampsia; the underlying mechanisms impacted by this comorbidity or how it may contribute to the development of preeclampsia remain unclear and warrant further study (Spradley et al., 2015b).

### Mouse models of obesity

Obesity is typically studied by feeding animals a diet high in fat to increase body weight (Christians et al., 2019). Masuyama and Hiramatsu studied the effect of obesity on pregnancy in adult, eight-week-old ICR mice after being fed a high-fat diet (HFD) consisting of 62% of calories from fat for 4 weeks (Masuyama and Hiramatsu, 2012). The prepregnancy weights of female ICR mice fed the high-fat diet were not reported; however, at the end of gestation, pregnant female high-fat diet mice were on average 16 g heavier than those on the control diet (Masuyama and Hiramatsu, 2012; Masuyama et al., 2016). High-fat diet pregnant mice exhibited increased insulin resistance with poor glucose tolerance, as well as increased serum levels of triglycerides and leptin, but had decreased levels of adiponectin (Masuyama and Hiramatsu, 2012; Masuyama et al., 2016).

Pregnant high-fat diet mice developed significantly elevated blood pressure at E18.5 accompanied by proteinuria (Masuyama and Hiramatsu, 2012). Also present was a significant increase in fetal weight, but no change in placental weight or litter size was observed (Masuyama and Hiramatsu, 2012; Masuyama et al., 2016). Placental morphology, lineage, immune, or angiogenic markers were not assessed in these studies (Masuyama and Hiramatsu, 2012). However, placental morphology was reported to be altered in a different study using pregnant obese mice (45% high-fat diet), where high-fat diet was associated with decreased labyrinth thickness, compared to the control diet (Kim et al., 2014).

A standard way to induce obesity in mice is to use a diet high in fat content (i.e., ∼60% of calories from fat). It should be noted; however, that this percentage of calories to induce obesity is not generally representative of typical Western diets, which is closer to ∼35–45% fat (Hintze et al., 2018). Also, not all strains of mice develop obesity on a high-fat diet, which suggests that an underlying genetic predisposition for obesity may be present in certain strains (Nishikawa et al., 2007; Huang et al., 2020; Li et al., 2020). Overall, the high-fat diet mouse model exhibits some of the classical hallmarks of preeclampsia, but further studies are required to better define inflammatory and angiogenic factors in these obese mice that develop preeclampsia-like symptoms.

## Mouse models with multiple comorbidites

### Blood pressure high/5 (BPH/5)

Work by Davisson et al., led to the identification of a mouse line, Blood Pressure High 5 (BPH/5), that is borderline hypertensive throughout adult life and spontaneously develops the hallmarks of preclampsia during pregnancy (Davisson et al., 2002). BPH/5 mice are a substantially inbred subline, derived from >20 generations of brother-sister matings of the more well-known, hypertensive BPH/2 strain (Schlager and Sides, 1997; Davisson et al., 2002). BPH/5 mice have significantly increased blood pressure prior to pregnancy, compared to C57BL/6 mice, as well as a significantly increased mean arterial pressure during pregnancy, beginning on gestational day 14 through parturition. The blood pressure of BPH/5 returned to the baseline “borderline hypertensive” levels after birth (Davisson et al., 2002). Endothelial dysfunction was also noted in pregnant BPH/5 mice and proteinuria as well as glomerulosclerosis were observed. In addition, fetal weights were significantly reduced and litter sizes were notably smaller (Davisson et al., 2002).

Analysis of BPH/5 pregnancies revealed that placental weights of BPH/5 placentas were similar to controls in late gestation (Dokras et al., 2006). All placental lineages were present; however, disruption of placental structure was noted, as trophoblast layers were disorganized and the expression of placental lineage markers were significantly reduced at E14.5 (Dokras et al., 2006). Vascular pathologies were also present in BPH/5 placentas, as blood spaces and branching morphogenesis were reduced and decidual vessels were characterized by thickened vessel walls, narrowed lumens, and increased uterine artery resistance (Dokras et al., 2006). Decreased VEGF and placental growth factor (PlGF) mRNA were observed in BPH/5 placentas at E10.5, while sFLT-1 mRNA was significantly increased, consistent with preeclampsia symptoms (Sones et al., 2018).

The BPH/5 mouse model presents with borderline hypertension and recapitulates several hallmarks of preeclampsia during pregnancy (Sones and Davisson, 2016). Disruption of normal placental development and smaller fetal weight at birth suggests that this model may be similar to early-onset preeclampsia with fetal growth restriction (Waker et al., 2021). Recent studies; however, have changed the context of the BPH/5 model and expanded the comorbidity beyond spontaneous chronic borderline hypertension and to now include preexisting metabolic disease, obesity, and fatty liver disease (Sutton et al., 2017; Reijnders et al., 2019; Johnston et al., 2021; Sones et al., 2021).

In addition, the reproductive axis in these mice is disrupted as the BPH/5 mice have reduced serum 17β-estradiol during estrous (Sutton et al., 2017). During pregnancy these mice continue to exhibit increased body weight and white adipose tissue with elevated cholesterol and triglyceride levels compared to C57BL/6 control mice, indicative of obesity (Reijnders et al., 2019). Caloric restriction ameliorated some of these pathologies and suggests that the BPH/5 phenotype might be linked to the metabolic disease, in addition to being chronically borderline hypertensive (Reijnders et al., 2019; Olson et al., 2020). The conjunction of these multiple comorbidities may make it challenging to determine the individual effects of chronic hypertension or obesity on the generation of preeclampsia. However, women that are both obese and hypertensive are a segment of the population at high risk of developing the condition and a mouse model that exhibits these preexisting conditions may be useful to study the interaction and impact of both comorbidities on the development of preeclampsia.

## Conclusion

Several mouse models with comorbidities that exhibit preeclampsia-like symptoms have been developed. These models vary in the techniques used to create them and how closely they recapitulate the human condition. Comorbidities such as chronic hypertension, pregestational diabetes, and obesity increase the risk of developing preeclampsia. As the prevalence of these conditions is predicted to increase in coming years, so too will the number of people that develop preeclampsia (Gortazar et al., 2020). The short-term economic costs of preeclampsia are in the billions and are even greater long-term, when the increased risk for chronic comorbidities for mother and offspring are also considered (Martin et al., 2011; Stevens et al., 2017; Burton et al., 2019; Rana et al., 2019; Waker et al., 2021).

Preeclampsia is a heterogenous condition that differs in timing and severity, indicating that many models may be needed to uncover underlying mechanisms that are common and disparate. As preeclampsia only spontaneously occurs in humans and a few higher apes, mouse models are well-accepted to model the condition preclinically. While several mouse models with preexisting comorbidities exist, not all are representative of the human condition, thus choosing which models to adapt to study preeclampsia is of importance.

Although autoimmune diseases (systemic lupus erythematosus, antiphospholipid syndrome) are significant risk factors for preeclampsia, no mouse models currently exist that investigate preeclampsia with these preexisting conditions prior to pregnancy. Autoimmune antibodies administered during a normal mouse pregnancy, such as antiphospholipid or agonistic angiotensin type 1 receptor autoantibodies, can induce preeclampsia-like symptoms; however, this does not model a preexisting condition. In addition, no reported mouse models of a previously preeclamptic pregnancy or preexisting chronic kidney disease, which are high risk factors, have been studied in relation to the development of preeclampsia. While gestational diabetes with subsequent onset of preeclampsia does occur in some patients, gestational diabetes generally only occurs after the first trimester of pregnancy and therefore is not a preexisting condition. Perhaps most challenging, but nonetheless important, are the lack of complex mouse models with multiple preexisting conditions often observed in the human population that are at high risk of developing preeclampsia; such as: obesity with diabetes, chronic hypertension with obesity, or chronic hypertension with obesity and diabetes.

There is an urgent need to increase the development of mouse models of preeclampsia with preexisting conditions that recapitulate the human patient population. The use of advanced techniques in combination with preexisting comorbidity models, such as trophoblast-specific gene transfer of HIF-1α in mice on a high fat Western diet or sFLT1 in non-obese diabetic mice, may more accurately reflect preeclampsia superimposed on a comorbidity (Kaufman et al., 2014; Albers et al., 2019; Vaughan et al., 2021). Future studies will improve our understanding of how preexisting conditions impact the development, timing, and severity of preeclampsia and will provide a much-needed foundation for subsequent preclinical and translational studies.
